# Scopolamine Hydrobromate

**Published:** 1894-09

**Authors:** Arthur G. Hobbs

**Affiliations:** Atlanta, Ga.


### SCOPOLAMINE HYDROBROMATE.

By ARTHUR G. HOBBS, M.D.,

Atlanta, Ga.

This new mydriatic is being tested now by some oculists, especially by those who are in charge of large clinics where it can be conveniently alternated with atropia, homatropia, etc., and the results compared. It seems at present to occupy a middle ground between atropia on the one hand, which is known to produce a complete paralysis of the accommodation and retain its effects for a week or ten days, and homatropia, which is claimed by some, and denied by others, to effect a complete control of the accommodative muscles and retain its effects only twenty-four hours. In the first case the time necessary to accomplish a full paralysis ranges from three to four days, and in the second only sixty to ninety minutes according to its advocates.

Scopolamine requires about two hours, with intervals of twenty minutes between the instillations, according to my observations, to bring about the desired result, and so far as I have been able to decide by comparative tests, the full effect is then reached quite as completely as that produced by a three days use of atropia. Its paralysis lasts from two to three days, so it occupies a place between these two leading mydriatics, both in the time of reaching its maximum effect and in the time of its complete decline. The above comparison is based upon the assumption of the advocates of homatropia that this drug produces a complete relaxation of the accommodative muscles in one to one and a half hours, the truth of which I am not yet fully prepared to accept. In the same proportion that atropia is used, in a one half to a one per cent. solution (approximately two to five grains to the ounce), and homatropia in a one per cent. solution (about four to five grains to the ounce), so scopolamine is used as a rule, in solutions of two to five grains to the ounce, although I prefer it in weaker solutions. It should be dropped into the eye at intervals of twenty minutes until four instillations have been made, when it may be assumed that complete paralysis of the accommodative muscles has been reached.

It has served me thus far in such refractive tests that have required complete paralysis of the accommodation quite as well as atropia, and while it does its work much more quickly, its decline is at most only about one-fifth and oftener one-sixth of the time required when atropia has been used. Homatropia will be the mydriatic, however, when we can become convinced that it effects a complete paralysis of the accommodation in one hour or even in twice that time, since there can be no question of its more rapid decline. Scopolamine produces no unpleasant effects in the throat, and it allows a possible accommodation within a day and a half or two days after its instillation, although the pupil may not become normal for a day or two longer.

Great comfort may be extended to the patient, particularly if he should be a business man with few hours to lose from his work, by prescribing a weak solution of eserine (one-eighth grain to the ounce) in order to more rapidly reduce the size of the pupil and indirectly aid the restoration of accommodation.

Some have asserted that scopolamine does not increase intraocular tension, but its use has not yet been sufficiently extensive to fully warrant this statement. Should this be true, scopolamine would possess a great advantage over atropia, especially in older subjects. In some phlyctenular forms of keratitis, as well as iritis, when occurring beyond middle age, it seems particularly efficacious; indeed I have already learned to place more confidence in it and feel safer when using it in such cases. Although it is much less expensive now than when I first began its use, about two years ago, its cost still restricts its general use.

We may hope that the consensus of opinion will in a year or two furnish us with a more accurate knowledge of this drug, to indicate to us when and in what cases it is preferable to atropia or homatropia, in case it should ever reach this point of usefulness.

It is my opinion that it will in many cases supersede the first, and entirely supplant the last, unless homatropia should prove itself equal to the occasion now claimed for it by many. In this latter case homatropia would supersede both atropia and scopolamine for refraction work, and leave a very narrow field of usefulness for the latter. Since the contingency still exists, however, we may continue to use scopolamine, particularly where we fear atropia, as well as in refraction work.

